# Pre- and during -COVID-19 pandemic mortality trends and drivers in rural, coastal Kenya: findings from the Kaloleni–Rabai Health and Demographic Surveillance System

**DOI:** 10.1186/s12963-025-00434-5

**Published:** 2025-12-02

**Authors:** Rosebella Iseme-Ondiek, Morris Ogero, Rachael Odhiambo, Beth Tippett Barr, Chodziwadziwa Kabudula, Jean J. H. Bashingwa, Anthony K. Ngugi

**Affiliations:** 1 Department of Population Health, Aga Khan University, Nairobi, Kenya; 2 London School of Hygiene and Tropical Medicine, London, England; 3 Institute for Human Development, Aga Khan University, Nairobi, Kenya; 4 Nyanja Health Research Institute, Salima, Malawi; 5 MRC/Wits Rural Public Health and Health Transitions Research Unit (Agincourt), Faculty of Health Sciences, School of Public Health, University of the Witwatersrand, Johannesburg, South Africa

**Keywords:** Mortality trends, COVID-19, Excess COVID mortality, Rural Kenya, Mortality surveillance, Community deaths, HDSS

## Abstract

**Background:**

There is contradicting information regarding the effect of COVID-19 on mortality in African settings. Knowledge of the complete direct and indirect burden of COVID-19 on mortality is heavily reliant on the availability of a population-based surveillance system. Here we provide robust data on the effect of COVID-19 on mortality trends in a rural, coastal, Kenyan community.

**Methods:**

A historical cohort study using data from the Kaloleni Rabai Health and Demographic Surveillance System was conducted with special focus on two discernible time periods representing the pre-COVID-19 (2018–2019) and COVID-19 (2020–2021) periods. Mortality rates were estimated as the total number of deaths divided by the person-time (years) at risk, accounting for attrition, and calculated separately for the two periods. A cox proportional hazards model was used to estimate the impact of COVID-19 on mortality.

**Results:**

1191 deaths occurred between 2018 and 2021. There was no significant change in overall mortality rates between pre-COVID-19 and COVID-19 periods (3.7 and 3.6 per 1000 person years at risk respectively, *p* = 0.74). Older age was significantly associated with mortality (a_HR: 1.05, 95% CI: 1.05–1.06; *p* < 0.001). However, an interaction term between age and time-period appeared to reverse this association (a_HR: 0.99, 95% CI: 0.99–1.00; *p* < 0.001).

**Conclusions:**

Our findings suggest that although overall COVID-19 did not directly impact mortality rates within this rural population, the onset of the pandemic did appear to reverse and/or attenuate the impact of several risk factors on mortality. It is possible that COVID-19 brought health and wellness into sharp focus, making people more vigilant about their health, hygiene and associated preventive measures.

## Background

Mortality statistics represent a valuable means of evaluating community health, and an important method to monitor and ultimately improve the health status of an entire community. As many causes of death are preventable or treatable, there are obvious benefits for populations where evidence-based public health prevention efforts are in place. In this regard, mortality data allow us to identify leading causes of premature death and provide a valuable benchmark for evaluating progress in increasing years of healthy life for a given population. They are therefore important indicators of where national, county, and sub-county prevention efforts should be placed in building healthy communities.

In December 2019, the world was introduced to the coronavirus disease 2019 (COVID-19), when reports of a pneumonia of unknown cause first emerged in Wuhan China [1, 2]. COVID-19, caused by the highly communicable severe acute respiratory syndrome coronavirus 2 (SARS-CoV-2) virus spread rapidly, and by early October 2023, over 771 million cases of COVID-19 had been confirmed in more than 194 countries, with almost 7 million COVID-19 related fatalities recorded [3]. It is important to note that during the pandemic, COVID-19 related deaths could have been attributed to other causes, particularly in countries where COVID-19 testing wasn’t widely available, potentially leading to an underestimation of COVID-19 related mortality. In this regard, other than deaths occurring directly because of the effects of exposure to SARS-CoV-2, COVID-19 may have exacerbated existing comorbid conditions, such as cardiovascular disease (CVD), diabetes, hypertension, and pulmonary disease [4]. COVID-19 has also been linked to the development of cardiovascular disease with subsequent occurrence of death because of the post-acute sequelae of COVID-19 [5, 6].

Notably, the appearance of the first COVID-19 case in several countries triggered both local and national governments across the globe, including in low-and-middle income countries to intervene and issue directions under executive orders aimed at containing further spread of the virus [7]. In Kenya, a range of non-pharmaceutical interventions (NPIs) aimed at limiting transmission of the virus were implemented [8]. These measures, as in other countries, included travel bans, cancellations of public gatherings, social distancing, school closures, recommendations to work from home and stay at home, and nationwide lockdowns. Importantly, in countries such as Kenya these measures were instituted with insufficient social support for disadvantaged populations from low socioeconomic standing. Consequently, COVID-19 related deaths during this period may also reflect deaths indirectly related to the pandemic, including deaths associated with reductions in access to health care, hospital avoidance due to fear of COVID-19 infection, increases in drug overdoses, and economic hardship leading to housing and food insecurity [9].

The human, economic and social cost of the COVID-19 pandemic on rural populations is unclear. In this regard, rural areas characterized by poor infrastructure, including few hospitals, healthcare facilities, and specialized medical services had a lower capacity to measure or mitigate the impact of the disease [10, 11]. The vulnerability associated with the absence of adequate infrastructure was further compounded by the reliance on self-employment as well as small or micro-businesses which were significantly affected by the financial constraints brought about by the pandemic and the measures implemented to control its spread [10–12]. Moreover, vaccination rates were lower in rural than in urban areas [13]. As such, an understanding of the true impact of the pandemic on rural communities is of importance. In particular, as developing countries (and rural communities in particular) are also often characterized by incomplete vital registration systems, population-based health and demographic surveillance systems provide an opportunity to accurately examine mortality trends within well-defined populations. This is important to note, as biases in efforts to understand the impact of the pandemic can lead to poor policy decisions and failed efforts to safeguard the health of populations in the face of emerging health risks.

Our study examined whether the COVID-19 pandemic years influenced changes in mortality trends and risk within a rural coastal setting in Kenya, using data from an existing health and demographic surveillance system. We additionally determined whether the effect of factors likely to influence mortality outcomes differed between the two time periods.

## Methods

### Study design

Our historical cohort study used data from 2018 to 2021. Specifically, our study focused on two discernible time periods representing the pre-COVID-19 period (2018–2019) and during-COVID-19 period (2020–2021).

### Characteristics of the study setting and its population

**Fig. 1:**
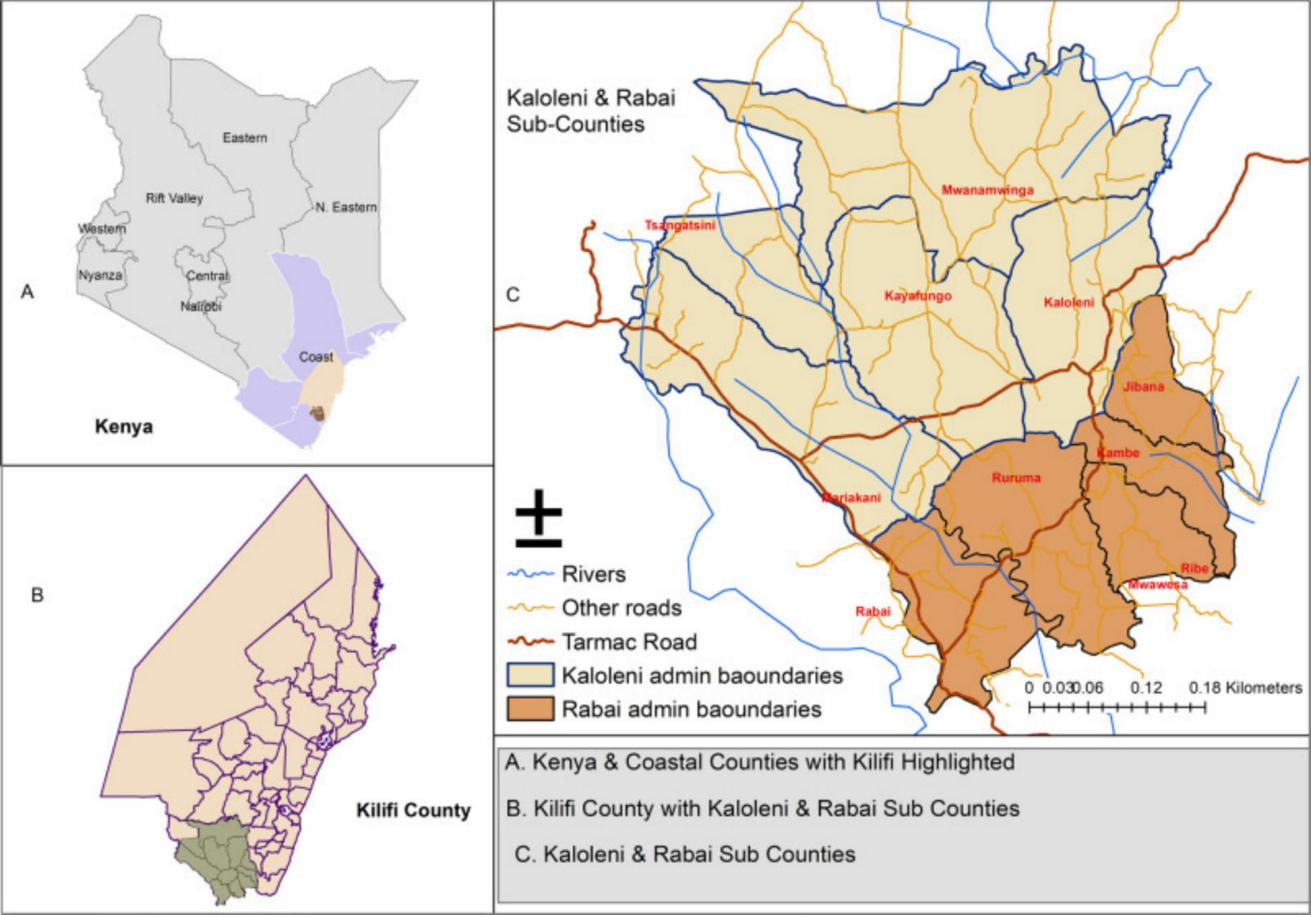
Map illustrating the Kaloleni/Rabai Health and Demographic Surveillance System (KRHDSS) site situated in Kilifi County [15]

The study area comprises two of the seven sub-counties in Kilifi County, one of five coastal counties in Kenya. Specifically, our study was situated in the south of Kilifi County, within the largely rural areas of Kaloleni and Rabai sub-counties. The two sub-counties cover an area of approximately 909 km^2^. They are made up of 10 community health units (CHUs) (and 114 villages) representing community-level health service delivery structures, each covering on average 1000 households, or 5000 people [14]. The population is predominantly Christian and Muslim. A map of the study area is presented in Fig. 1 [15]. Kenya’s four other coastal counties are shown in purple, with Kilifi highlighted in beige in Fig. 1A. Kaloleni and Rabai subcountries are located at the southern tip of Kilifi County, marked in the darker shade in Fig. 1A and B. The map of the 2 sub-counties appears exploded in Fig. 1C.

Both Kaloleni and Rabai constitute areas within the county (and the larger country as well) with low socioeconomic status; approximately 70% of the population live below the poverty line [16, 17]. Economic activities in the area include subsistence agriculture, crafts, casual labour, and informal trading, and high net migration into the area is the predominant driver of population growth [15]. Notably, the major contributors of morbidity within our study area are infectious and parasitic diseases including malaria, schistosomiasis, and HIV related disease [18]. Injuries are the leading cause of hospital admissions among men, while diabetes ranks as the third leading contributor to years lost in both life expectancy and quality of life, as measured by Disability-Adjusted Life Years (DALYs), followed by stroke [18].

Following Kenya’s first confirmed COVID-19 case on the 13th of March 2020 (and Kilifi’s first confirmed case on 22nd March 2020), several restrictions were implemented to curb the spread of the disease-causing virus SARS-CoV-2 [19–21]. Firstly, a mandatory quarantine for all passengers arriving on international flights was immediately instituted (15th March). Thereafter, on 20th March all learning institutions were closed and 5 days later (25th March) all international passenger flights were banned, and a nationwide curfew was implemented from dusk until dawn (19 h 00 to 05 h 00) except for individuals providing essential and critical services such as healthcare workers. On April 6th, restrictions were imposed on the number of passengers allowed in public and private transport vehicles. New rules for ferry operations were also introduced, i.e., all passenger and private vehicles were limited to 50% capacity, ferry services were restricted to specific hours, and ferries carrying passengers were prohibited from carrying cargo. Additionally, mandatory mask-wearing in public places was implemented, and travel in and out of four counties, including Kilifi, was restricted by road, rail, or air, except for emergency services and cargo transportation. Notably, Kilifi only received its first consignment of COVID-19 vaccines in March 2021, and significant challenges were noted in vaccine administration as a result of the County’s vast terrain and under staffed health facilities [22]. Between April 2021 and August 2022, Kilifi County had registered a total of 5198 cases. By March 2022, the County had administered only 273,306 out of 296,903 available doses of various types, serving a population of over 1.5 million people [22]. Due to the nationwide lockdown and restricted travel into Kilifi County, which had one of the highest case numbers, the regular bi-annual population enumeration in Kaloleni and Rabai sub-counties as part of the Kaloleni–Rabai Health and Demographic Surveillance System (KRHDSS) was disrupted. As a result, data collection shifted to an annual schedule, taking place from July to December. It is however important to note that data collection was still undertaken in-person during routine household health promotion visits, in line with the approved surveillance protocol and alongside recommended COVID-19 protocols, for the safety of both the enumerators and respondents.

### Study participants and study size

Our study comprised the entire population of Kaloleni and Rabai sub-counties in Kilifi County, amounting to 125,577 individuals who were residents for at least 6 months between 2018 and 2021. As part of the Health and Demographic Surveillance System, all residents (except for 51 households) provided verbal consent and were enumerated [15]. Due to missing data, a total of 125,393 individual records (representing 22,258 households) were used in our study.

### Data sources and measurements

We used data from the existing Kaloleni–Rabai Health and Demographic Surveillance System (KRHDSS). KRHDSS is a population and health information registry developed collaboratively by Aga Khan University (Department of Population Health) and the local health authorities of Kaloleni and Rabai sub-counties in the coastal area of Kilifi County, Kenya. This system is integrated within the local community health infrastructure and consistently captures essential demographic and health information, vital status, and migration patterns within the local community. The KRHDSS was established in the first half of 2017, where approximately 30% of the population of the Kaloleni and Rabai sub-counties (approximately 78,000 individuals) was registered and enumerated and their baseline health and demographic status recorded. This platform is described elsewhere (15) (18) but briefly, data are collected electronically by community health volunteers (CHVs) using tablets during their routine health promotion and education activities in households, in line with the Kenya government community health strategy (CHS). CHVs receive annual refresher training on data collection tools and procedures prior to every new round of data collection, delivered by members of the Department of Population Health, Aga Khan University, and the local County Health department. Supervision of data collection as well as on-field support to CHVs is similarly undertaken by members of the two organizations. Initially (2017–2019), data were collected during biannual update rounds, and annually during COVID-19. The system collects information on a range of demographic and vital events, maternal and child health, water, sanitation, and hygiene indicators [23]. The indicators and their definitions are based on the Ministry of Health household-level data collection tools for monitoring community health workers during their routine household visits [24]. From the field, the data are synced directly to a central server domiciled at Aga Khan University’s information and communication technologies (ICT) department. Each individual resident and household within the KRHDSS have been assigned a unique identification number that is used to link their data and enable tracking of their unique attributes across data collection rounds.

### Data management

Data from six rounds of data collection (specifically, January–June 2018; July–December 2018; January–June 2019; July–December 2019; July–December 2020; July–December 2021) were merged into a single dataset based on a unique individual identifier. The dataset underwent deduplication and data cleaning procedures were implemented to ensure the consistency of chronological transitions associated with individual vital events, including births, outmigration, immigration, and deaths.

A script was developed using the Julia programming language to identify and flag records with inconsistent documentation of events and dates [25]. Any identified data inconsistencies were then forwarded to the data manager for verification and correction.

For each individual, person-years were computed by calculating the difference between the entry date into the HDSS and either the date of outmigration or death. The resulting duration was divided by 365.25, taking into account leap years. In subsequent statistical analyses, person-years were employed to account for varying durations of exposure.

### Statistical analysis

In the current analyses, the outcome of interest was all-cause mortality. We characterized all-cause mortality rates and trends, by age group (0–4 years, 5–14 years, 15–29 years, 30–54 years, 55+ years), sex and period [pre- (2018–2019) and during-COVID-19 (2020–2021)]. Mortality rates were estimated as the total number of deaths divided by person-years at risk, accounting for time lost due to attrition, calculated separately for the two periods. Chi-square test was used to test for associations between mortality and time-period for various factors. To estimate the impact of COVID-19 on mortality risk we performed a survival analysis (i.e., cox proportional hazards model). Prior to survival modelling we used Lexis expansion to disaggregate the age of each participant across their observation period. This means that each participant’s follow-up time was divided into intervals based on their age at each point in time. This allowed us to account for the influence of age as a time-varying factor on mortality risk. The survival models adjusted for the potential influence of factors known to impact morbidity and mortality risk within the study population, including (1) age, (2) sex, (3) access to health care (distance to health facility), (4) water, sanitation and hygiene (access to safe water, treatment of drinking water, ownership of a functional latrine, ownership of handwashing equipment, ownership of a refuse disposal facility), (5) malaria risk (use of long-lasting insecticide-treated nets), (6) travel time to the nearest health facility in minutes, (7) presence of self-reported non-communicable conditions or disabilities, and (8) household size. Detailed description and definitions of the latter variables are provided by Ngugi et al. [23]. We used AccessMod software V5 [26, 27] to estimate travel time by creating a travel impedance surface. This involved combining land cover, elevation, and road network data to generate a travel impedance raster surface, then assigning travel speeds based on different transport modes. Land cover data helped determine travel speeds for various terrain types, while elevation data accounted for landscape changes that affect travel time. Road network data was used to find the shortest route between two points. Travel time was then estimated by using the geographical coordinates of each household, the location of health facilities, and the generated raster file. Ogero et al. provides a detailed description of the approach used to estimate travel time to nearest health facility within this study population [28]. All analyses were undertaken using R Statistical Software (v4.3.0) [29].

### Ethical considerations

Data collection and use of secondary data from the KRHDSS database had ethical approval from the Aga Khan University Institutional Scientific and Ethics Review Committee (2013/IREC-65(v6); 2013/IREC-65(v7); 2013/IREC-65(v8); 2013/IREC-65(v9); 2013/IREC-65(v10)). No identifiable information was used in this analysis.

## Results

Between 2018 and 2021 our study population had a total of 125,393 individuals residing in 22,258 households. Over one quarter of the study population were aged between 15 and 29 years of age (29.0% of the pre-COVID, and 28.5% of the COVID-19 populations), and over half were female (51.9 and 51.2%, respectively). Similarly, across both periods, more than half of the population treated their drinking water and owned functional latrines and refuse disposal facilities. Residents travelled between 17 and 62 min to reach the nearest health facility, with the average 43 min travelled to the nearest health facility remaining constant between the pre and during COVID-19 periods.

There were significant changes between the two time periods in the proportion of the population aged 0–4 years, from pre-COVID-19 at 17.4% (95% CI: 17.1–17.7), and in the COVID-19 period at 15.6% (95% CI: 15.3–15.9), and in those aged 30–54 years, who comprised 18.4% (95% CI: 18.1–18.7) in the pre-COVID period, and 20.4% (95% CI: 20.1–20.7) during COVID-19 (Table 1). During COVID-19, ownership of handwashing equipment declined by 4% compared to the pre-COVID period, despite a 2–5% increase in the proportion of the population with access to other water and sanitation facilities (safe water, treated drinking water, functional latrines, and refuse disposal facilities).

**Table 1:** Sociodemographic characteristics of study population by period (pre and during COVID-19)

	Population		*p* value
	Pre-COVID-19 (2018–2019)	During-COVID-19 (2020–2021)	
Age group			
0–4 years	12,046 (17.4%)	8761 (15.6%)	**<0.001**
5–14 years	18,064 (26.1%)	14,608 (26.0%)	0.744
15–29 years	20,057 (29.0%)	16,025 (28.5%)	0.089
30–54 years	12,762 (18.4%)	11,458 (20.4%)	**<0.001**
55+ years	6301 (9.1%)	5311 (9.5%)	**<0.001**
Sex			
Female	35,955 (51.9%)	28,767 (51.2%)	**<0.001**
Male	33,275 (48.1%)	27,396 (48.8%)	**<0.001**
Home health and hygiene			
Access to safe water	30,777 (44.3%)	26,040 (46.4%)	**<0.001**
Treatment of drinking water	40,176 (57.9%)	34,257 (61.0%)	**<0.001**
Ownership of a functional latrine	47,821 (68.9%)	40,974 (73.0%)	**<0.001**
Ownership of handwashing equipment	33,125 (47.7%)	24,132 (43.0%)	**<0.001**
Ownership of a refuse disposal facility	37,255 (53.7%)	31,128 (55.4%)	**<0.001**
Use of insecticide treated nets	60,720 (87.5%)	51,108 (91.0%)	**<0.001**
Travel-time to the nearest facility			
Median; IQR	43.2 (40.1–43.5)	43.3 (43.1–43.3)	
<1 h	64,326 (92.7%)	52,578 (93.6%)	**<0.001**
Self-reported NCDs or disability	814 (1.2%)	560 (1.0%)	0.003
Household size			
Median; IQR	7 (5–10)	7 (5–9)	
≤4 members	12,836 (18.5%)	13,011 (23.2%)	**<0.001**

*NCD* non-communicable disease, *IQR* Interquartile range, <0.001 indicates less than 0.1% probability of observing the study results by chance

There were 69,230 individuals who contributed 168,074 person years at risk in the pre-COVID-19 period, and 56,163 individuals who contributed 158,392 person years at risk during COVID-19. A total of 1190 deaths occurred in the study population between 2018 and 2021 (619 before COVID-19, and 571 during). Of the determinants of mortality presented in Table 2, only sex, age, household size, ownership of a refuse disposal facility, use of insecticide treated nets, having a NCD or disability and travel time to the nearest health facility were observed to have a statistically discernible association with mortality. Whilst access to a refuse disposal facility (hazard ratio = 1.15; (1.052–1.258); *p* = 0.002), male sex (hazard ratio = 1.46; (1.334–1.598); *p* = <0.0001), older age (hazard ratio = 1.048; (1.046–1.05); *p* = <0.0001) and having an NCD or disability (hazard ratio = 4.931; (3.986–6.1); *p* = <0.0001) were noted to increase the risk of mortality, use of insecticide treated nets (hazard ratio = 0.794; (0.717–0.88); *p* = <0.0001), a household size of greater than 4 members (hazard ratio = 0.935; (0.922–0.948); *p* = <0.0001), and travel time to a health facility greater than 1 h (hazard ratio = 0.759; (0.611–0.942); *p* = <0.012), showed a protective effect. The presence of a NCD or disability was observed to confer the largest increase in mortality risk (hazard ratio = 4.931; (3.986–6.1); *p* = <0.0001) within this population between 2018 and 2021. Noticeably, access to safe water, treatment of drinking water as well as ownership of a handwashing facility or functional latrine did not appear to have any direct bearing on mortality within this population. Overall, there was no discernible difference in mortality risk when comparing the pre- and during COVID-19 periods (hazard ratio = 1.26E−09; (1.3E−297–1.2E+279); *p* = <0.952) (Refer to Table 2).

**Table 2:** Hazard ratios for mortality risk factors: univariable cox regression analysis

Variable	Hazard ratio (HR)	Lower 95% CI	Upper 95% CI	*p* value
Sex (male)	1.46	1.334	1.598	<0.0001****
Safe water access (yes)	0.958	0.876	1.048	0.347
Age	1.048	1.046	1.05	<0.0001****
Water treatment (yes)	0.956	0.874	1.046	0.33
Latrine use (yes)	0.987	0.899	1.085	0.791
Handwashing facility (yes)	1.013	0.92	1.116	0.788
Refuse (yes)	1.15	1.052	1.258	0.002*
Use of LLINs (yes)	0.794	0.717	0.88	<0.0001****
COVID phase 1 (2020–2021)	1.26E−09	1.3E−297	1.2E+279	0.952
NCDs/disability (yes)	4.931	3.986	6.1	<0.0001****
Household size (4+)	0.935	0.922	0.948	<0.0001****
Travel time > 1 h	0.759	0.611	0.942	0.012*

*LLINs* long lasting insecticide treated nets, *NCDs* non-communicable diseases, *CI* confidence interval

* *p* < 0.05; ** *p* < 0.01; *** *p* < 0.001; **** *p* < 0.0001

The multivariable regression analyses presented in Table 3 reveals that age, sex, access to a functional latrine, NCD comorbidity and travel time to the nearest health facility were the only variables to exhibit a statistically significant association with mortality when all risk factors including onset of COVID-19 were included in the multivariable model. The risk of mortality was observed to increase in parallel with increasing age (adjusted hazard ratio = 1.05; (1.05–1.06); *p* = <0.001), male gender (adjusted hazard ratio = 1.66; (1.42–1.95); *p* = <0.001), having a NCD/Disability (adjusted hazard ratio = 2.64; (1.71–4.09); *p* = <0.001), travelling more than 1 h to the nearest health facility (adjusted hazard ratio = 1.76; (1.20–2.57); *p* = 0.004), and having access to a functional latrine (adjusted hazard ratio = 1.35; (1.12–1.63); *p* = 0.002).

**Table 3:** Hazard ratios for mortality risk factors: multivariable cox regression analysis

Variable	Hazard ratio (exp(β))	95% CI (lower)	95% CI (upper)	*p* value
Sex (male)	1.66	1.42	1.95	<0.001***
Age	1.05	1.05	1.06	<0.001***
Safe water access (yes)	1.15	0.93	1.43	0.199
Water treatment (yes)	0.96	0.76	1.20	0.698
Latrine use (yes)	1.35	1.12	1.63	0.002**
Handwashing facility (yes)	0.83	0.68	1.02	0.081
Refused (yes)	1.11	0.92	1.34	0.261
Use of LLINs (yes)	1.01	0.83	1.22	0.941
COVID phase 1 (2020–2021)	2.48 × 10^−9^	NA	NA	0.955
NCDs (yes)	2.64	1.71	4.09	<0.001***
Household size (4+)	0.82	0.68	0.98	0.032*
Travel time > 1 h	1.76	1.20	2.57	0.004**
Sex (male)^a^ COVID-19 period	0.96	0.79	1.17	0.712
Age^a^ COVID-19 period	0.99	0.99	1.00	<0.001***
Household Size (4+)^a^ COVID-19 period	0.94	0.75	1.16	0.553
Use of LLINs^a^ COVID-19 period	0.76	0.60	0.95	0.018*
Safe water access^a^ COVID-19 period	0.84	0.64	1.09	0.189
Water treatment^a^ COVID-19 period	1.04	0.79	1.37	0.793
Handwashing facility^a^ COVID-19 period	1.17	0.92	1.49	0.210
Refused (yes)^a^ COVID-19 period	1.15	0.92	1.43	0.226
Latrine use^a^ COVID-19 period	0.65	0.52	0.81	<0.001***
NCDs/disability (yes)^a^ COVID-19 Period	1.07	0.65	1.77	0.793
Travel time > 1 h^a^ COVID-19 period	0.59	0.37	0.93	0.024*

*LLINs* long lasting insecticide treated nets, *NCDs* non-communicable diseases

* *p* < 0.05; ** *p* < 0.01; *** *p* < 0.001; **** *p* < 0.0001

^a^Interaction term for COVID-19 period, 2020–2021

## Discussion

We examined mortality trends and determinants within a well-defined rural, coastal population in Kenya. In particular, we sought to determine whether the onset of COVID-19 directly or indirectly impacted mortality by comparing the period immediately prior to the onset of COVID-19 (2018–2019) to the period following its emergence (2020–2021). While the COVID-19 period itself did not appear to increase all-cause mortality, the pandemic did appear to modify the effect of other risk factors on mortality. For example, where a statistically significantly higher risk of mortality was noted amongst males when compared to their female counterparts (multivariable analyses presented in Table 3), an interaction term between sex and time-(COVID-19) period appeared to attenuate this association. The higher mortality risk observed among males in the univariable analyses is consistent with national data showing that men accounted for the majority (65%) of confirmed cases in Kenya [30–33]. In addition, males have a higher prevalence of lifestyle factors that predispose to severe COVID-19 outcomes such as smoking of tobacco [34, 35]. Additionally, biological factors may play a role: males are thought to have a weaker immune response due to both genetic and hormonal factors [36]. Notably, males are also reported to have higher levels of angiotensin-converting enzyme2 (ACE2) receptors on the endothelium of the pulmonary vessels which facilitate binding of ACE2 by SARS-CoV-2, enabling viral entry into cells, possible exacerbating disease severity [37]. The binding of SARS-CoV-2 to ACE2 can lead to dysregulation of the angiotensin system leading to loss of ACE2 mediated protection (including homeostatic functioning of key organs such as the lungs, heart, and vasculature) as well as promotion of a proinflammatory cytokine response (the cytokine storm being characteristic of severe COVID-19 infection and disease progression) [38, 39]. Interestingly, it is possible that the pandemic led to behavioural and environmental changes that mitigated these risks. Heightened health awareness, along with infection control measures such as curfews and the closure of bars and pubs, may have promoted healthier lifestyles among men in our study population, potentially contributing to the observed attenuation of the sex-based mortality difference during the pandemic.

Notably, the onset of the COVID-19 pandemic appeared to modify the association between age and mortality. While both univariable and multivariable analyses showed an increased risk of mortality with advancing age, the inclusion of an interaction term for the COVID-19 period revealed a reversal of this trend—indicating a slightly reduced mortality risk per additional year of age during the pandemic. This finding aligns with observations from a study in Kenya’s Coastal region, where significant excess mortality was reported only among individuals aged 5–14 and 15–44 years during wave 1 (1st April 2020 to 4th October 2020: Wild-type strain), whereas excess mortality among adults aged 45–64 and ≥65 years was notably lower [21]. However, in the same population, mortality rates among older adults (65+ years) rose markedly in subsequent waves surpassing those of their younger counterparts (Wave 2—Wild Type: 5th October 2020 to 14th February 2021; Wave 3—Beta Alpha: 15th February 2021 to 4th June 2021; Wave 4—Delta: 5th June 2021 to 11th December 2021; Wave 5—Omicron BA1: 12th December 2021 to 16th April 2022). It is plausible that a similar shift in mortality pattern occurred in our study population, but with a delayed manifestation potentially due to the predominantly rural and remote nature of the area, which may have affected the timing and extent of COVID-19 transmission.

The US Centers for Disease Control and Prevention (CDC) recognizes age as the strongest known risk factor for severe COVID-19 outcomes [24, 25]. However, there is a need to consider not just the isolated effect of age on mortality but the age dependency of comorbidities, which are acknowledged to influence the course of COVID-19 disease [40, 41]. In fact, the risk of severe outcomes is increased substantially in people of all ages with NCDs, conditions which in sub-Saharan Africa are reported to develop at an earlier age than in the global north (≈55 years) [42–45]. Whether these ailments themselves contribute specifically to SARS-CoV-2 pathogenesis or whether they are simply indicative of biological age characterized by a dysregulated immune system (characterized by poor host protection or a dysfunction in either or both signaling and/or effector pathways and subsequent chronic inflammation), or a combination thereof remains unclear [46]. It is important to highlight that less than 10% of the study population was aged above 55 years and an even smaller proportion of the population (less than 2%) was noted to have a self-reported non-communicable disease or disability. It is possible that the low representation of older adults and individuals with underlying health conditions (i.e., few cases) may have limited the statistical power to detect a statistically significant increase in mortality risk amongst those with NCD/Disabilities, contrary to findings from other studies (Table 3). Diagnosis of NCDs in sub-Saharan Africa is also hindered by several factors such as poor access to health facilities and limited infrastructure including diagnostic resources [47]. Consequently, the majority of NCDs are often undiagnosed, possibly contributing to our inability to observe the reality of increased mortality with NCDs, i.e., misclassification resulting from inaccurate self-reporting could obscure the real impact of NCDs on mortality making it more difficult to detect a significant association.

Apart from age, only the effects (on mortality) of insecticide-treated bed net use, ownership of a functional latrine, and travel time greater than 1 h to a health facility, were significantly associated with mortality, after accounting for potential changes in these relationships during the COVID-19 phase. As with age, the onset of COVID-19, reversed the impact of LLIN use, access to a functional latrine and travel time on mortality—factors that were previously noted to increase mortality risk appeared to confer a protective effect during the COVID-19 period (refer to Table 3). In this regard, studies in Africa and Asia have demonstrated that the wealth of a household is amongst the key factors associated with household access to improved or basic amenities including WASH services [48–52]. Consequently, it is possible that access to resources such as a functional latrine and insecticide treated bed nets may very well be a proxy of neighborhood characteristics such as affluence [53]. As such our observations are consistent with a recent literature review, which reported that majority of studies included (, i.e., 86/95) found significantly higher COVID-19 mortality in areas of social disadvantage than in affluent areas [54]. It is important to highlight that the relationship between deprivation and risk of COVID-19 mortality is multifaceted and could be explained through multiple related pathways, i.e., increased vulnerability resulting from a higher burden of key clinical risk factors for adverse COVID-19 outcomes and/or increased susceptibility resulting from weakened resilience due to repeated/long-term exposure to social determinants of health such as adverse psychosocial circumstances that increase immunosuppression [54].

Similarly, population density is believed to reflect the potential for a greater number of interactions capable of achieving COVID-19 infection. Indeed, a recent ecological study reported a positive correlation between population density and COVID-19 incidence and mortality across counties of Northeast Brazil [55]. This may help explain the lower observed COVID-19 mortality risk among individuals living more than an hour away from the nearest health facility—likely indicative of a greater distance to an urban centre.

Our study had some limitations. Firstly, the emphasis on quantitative data while valuable for identifying trends limits the ability to further explore and explain the underlying relationships between variables, leaving important nuances and contextual factors unaddressed. Also, in addition to the absence of information on household socioeconomic status that would have allowed us to better understand some of our observed results, data on some of the important variables such as non-communicable diseases are self-reported. This creates potential for information bias within our study. However, as CHVs who are responsible for data collection are also trusted members of this community they are knowledgeable of major events within this study population as well as likely to encourage honest responses from the participants. In addition, data quality is enhanced through real-time web-based data checking with prompt feedback to field supervisors for corrective action. While we included mortality data from January 2020, even though COVID-19 was not confirmed in Kenya (and Kilifi) until March 2020, we acknowledge that this may introduce some inaccuracies in attributing early 2020 deaths to COVID-19. However, this approach aligns with global frameworks and other studies in Kenya [21, 56]. Additionally, the lack of data on the exact timing of deaths limits our ability to exclude early deaths. Finally, unlike other studies, we only considered deaths up to December 2021. It is possible that the absence of substantial evidence indicating a COVID-19 impact on the mortality for this rural population is because its effects may not have manifested as noticeable changes in mortality patterns within the observed time frame, i.e., 2 years.

## Conclusions

Overall, our study findings indicate the absence of a statistically significant direct effect of COVID-19 on mortality within our study population. This suggests that, at least in our sample, COVID-19 may not have directly contributed to a notable increase in deaths during the study period. Previous research has reported a heterogeneous impact of COVID-19 on mortality across different regions of Kenya [21, 56] highlighting variable effects of the pandemic on the country’s population. Notably, the onset of the pandemic did influence the relationship between a number of potential risk factors and mortality. Therefore, while COVID-19 itself may not have had a discernible direct effect, the pandemic’s broader socio-economic and health impacts likely played a role in shaping mortality patterns during the study period.

Importantly, not all countries have the infrastructure and capacity to register and accurately report all deaths. In fact, a lot of African countries lack viable nationwide systems capable of accurately tracking and recording vital events including deaths within their population and the situation is no different in Kenya. Consequently, HDSS-data provides a feasible opportunity to develop a valid profile of the mortality trends within well-defined populations and evaluate the population-level impact of different health events including disease outbreaks. This is particularly useful for rural settings that are often even more resource limited than their urban counterparts. Given that Kenya is predominantly rural, the approaches utilized within our study as well as the study findings have major implications for efforts aimed at generating the data needed to understand mortality and perhaps inform evidence-based interventions within our setting.

## Data Availability

Any interested parties can apply directly to the Kaloleni Rabai Health and Demographic Surveillance System Governance Committee to access the data used in this paper by contacting them at nbi.krhdss-dph1@aku.edu.

## Abbreviations

95% CI: 95% Confidence interval
AKU-ISERC: Aga Khan University Institutional Scientific and Ethics Review Committee
A_RR: Adjusted rate ratio
CVD: Cardiovascular disease
COVID-19: Coronavirus disease 2019
CHS: Community health strategy
CHU: Community health unit
CHVs: Community health volunteers
F: Females
ICT: Information and communication technologies
IQR: Interquartile range
KRHDSS: Kaloleni and Rabai Health and Demographic Surveillance System
M: Males
N: No
NCDs: Noncommunicable diseases
SARS-Cov-2: Severe acute respiratory syndrome coronavirus 2
WASH: Water. Sanitation and Hygiene
Y: Yes
